# A modified TNM staging system for non-metastatic colorectal cancer based on nomogram analysis of SEER database

**DOI:** 10.1186/s12885-017-3796-1

**Published:** 2018-01-08

**Authors:** Xiangxing Kong, Jun Li, Yibo Cai, Yu Tian, Shengqiang Chi, Danyang Tong, Yeting Hu, Qi Yang, Jingsong Li, Graeme Poston, Ying Yuan, Kefeng Ding

**Affiliations:** 1 Department of surgical oncology, and The Key Laboratory of Cancer Prevention and Intervention, Second Affiliated Hospital, China National Ministry of Education, Zhejiang University School of Medicine, No. 88 Jiefang Road, Hangzhou, Zhejiang Province 310009 China; 2 Engineering Research Center of EMR and Intelligent Expert System, Ministry of Education, Collaborative Innovation Center for Diagnosis and Treatment of Infectious Diseases, College of Biomedical Engineering and Instrument Science, Zhejiang University, Hangzhou, 310009 China; 3 Department of Surgery, School of Translational Studies, University of Liverpool, Aintree University Hospital, Liverpool, L9 7AL UK; 4 Department of medical oncology, and The Key Laboratory of Cancer Prevention and Intervention, Second Affiliated Hospital, China National Ministry of Education, Zhejiang University School of Medicine, No. 88 Jiefang Road, Hangzhou, Zhejiang Province 310009 China

**Keywords:** Colorectal cancer, TNM stage, Nomogram, Prognosis prediction

## Abstract

**Background:**

To revise the American Joint Committee on Cancer TNM staging system for colorectal cancer (CRC) based on a nomogram analysis of Surveillance, Epidemiology, and End Results (SEER) database, and to prove the rationality of enhancing T stage’s weighting in our previously proposed T-plus staging system.

**Methods:**

Total 115,377 non-metastatic CRC patients from SEER were randomly grouped as training and testing set by ratio 1:1. The Nomo-staging system was established via three nomograms based on 1-year, 2-year and 3-year disease specific survival (DSS) Logistic regression analysis of the training set. The predictive value of Nomo-staging system for the testing set was evaluated by concordance index (c-index), likelihood ratio (L.R.) and Akaike information criteria (AIC) for 1-year, 2-year, 3-year overall survival (OS) and DSS. Kaplan–Meier survival curve was used to valuate discrimination and gradient monotonicity. And an external validation was performed on database from the Second Affiliated Hospital of Zhejiang University (SAHZU).

**Results:**

Patients with T1-2 N1 and T1N2a were classified into stage II while T4 N0 patients were classified into stage III in Nomo-staging system. Kaplan–Meier survival curves of OS and DSS in testing set showed Nomo-staging system performed better in discrimination and gradient monotonicity, and the external validation in SAHZU database also showed distinctly better discrimination. The Nomo-staging system showed higher value in L.R. and c-index, and lower value in AIC when predicting OS and DSS in testing set.

**Conclusion:**

The Nomo-staging system showed better performance in prognosis prediction and the weight of lymph nodes status in prognosis prediction should be cautiously reconsidered.

**Electronic supplementary material:**

The online version of this article (10.1186/s12885-017-3796-1) contains supplementary material, which is available to authorized users.

## Background

The existing 7th edition American Joint Committee on Cancer (AJCC) tumor-node-metastasis (TNM) staging system is widely used to predict survival for colorectal cancer patients and to guide adjuvant chemotherapy after potentially curative surgery. The next edition would probably be applied next year, but the preview showed no change in the strategy to classify non-metastatic patients. The TNM staging system classifies patients with positive lymph nodes (N^+^) into stage III, regardless of T stage. However, patients with early T stage who are N^+^ can have better outcomes than high T stages N^−^ (negative lymph nodes) patients [1–3]. This phenomenon is called survival paradox and may mislead oncologists to overestimate the prognosis risk of stage IIIa but underestimate stage II.

Our former research established the T-plus staging system by re-analyzing the summary data from the Surveillance, Epidemiology, and End Results (SEER) tumor registry [4]. The relative weights of T stage and N stage were calculated based on their impact on survival. This study showed that T stage affected postoperative survival more significantly than N stage in non-metastatic colorectal cancer, and the survival paradox was also eliminated by adopting the T-plus staging system [4]. This staging system was verified by a Chinese cohort with 25-year follow-up [5]. However, the implemented process of this research applied linear regression, while the effect of TN combinations on survival may be non-linear. Hence, a more scientific method was required to address this problem.

Nomogram is a statistical method that incorporate multiple variables and reduce statistical predictive models into a single numerical estimate of the probability of an event [6]. Nomogram is widely used in predicting tumor prognosis [7]. Memorial Sloan Kettering of Cancer Center offered a nomogram system for colorectal cancer patients to estimate overall survival and disease free survival after surgery on its official website [8]. It was based on 128,853 patients with primary colon cancer reported to SEER in 2011 [9]. Su [10] verified the MSKCC nomogram system providing more accurate survival predictions than the 7th edition TNM staging system in an external Chinese cohort.

The aim of this study was to further verify the concept enhancing the weighting of T stage by constructing a modified TNM staging system for non-metastatic colorectal cancer based on nomogram analysis of individual data from SEER database.

## Methods

### Patients

Total 115,377 patients were enrolled from the SEER 18 Registries Research Data, November 2015 submission (1973–2013). All patients were diagnosed with colorectal cancer between 2004 and 2013 by histopathological examination.

The inclusion criteria were: (a) Primary site of tumor was colon (c18.0-c18.9, c19.9) or rectum (c20.9); (b) Histologic types were adenocarcinoma (8140), mucinous adenocarcinoma (8480), mucin-producing adenocarcinoma (8481), mucinous cyst-adenocarcinoma (8470), signet ring cell carcinoma (8490) or undifferentiated carcinoma (8010, 8020, 8021); (c) No distant metastasis (CS mets at dx: 00); (d) No other malignant tumor history (sequence number: 00).

The exclusion criteria were: (a) Survival was unknown or less than 3 months; (b) Site specific surgery was unknown (blank); (c) Not receiving surgery (Rx Summ--Surg Prim Site: 0); (d) Regional nodes examined was none or unknown (95–99); (e) Regional nodes positive was none or unknown (95–99); (f) Tumor destruction; no pathologic specimen or pathologic specimen unknown (Rx Summ--Surg Prim Site: 10–19) or no lymph nodes examined (Regional nodes examined: 0); (g) Unknown if surgery performed (Rx Summ--Surg Prim Site: 99) or with no lymph nodes examined (Regional nodes examined: 0). Abbreviations complied with the Coding and Staging Manual of SEER [11].

Total 1194 patients were enrolled from a database of the Second Affiliated Hospital of Zhejiang University (SAHZU) for external validation. All patients were diagnosed with colorectal cancer between 2005 and 2011 by histopathological examination. Detailed and sufficient pathological and surviva information was extracted. Exclusion criteria were death by surgical complications within a 3-month postoperative period, stage 0 or stage IV disease, multiple colorectal cancer, or prior history of malignancy.

### Database cleansing

The basic patient data from SEER and SAHZU database included age, gender, race, follow-up months, survival status, invasive depth of tumor (T stage) and the number of involved lymph node (N stage), etc. Three different survival phases (1-year, 2-year and 3-year) were extracted according to 25 combinations of T stage (1 = T1, 2 = T2, 3 = T3, 4 = T4a, and 5 = T4b) and N stage (0 = N0, 1 = N1a, 2 = N1b, 3 = N2a, and 4 = N2b). Stage N1c (tumor deposit) were classified as N1b considering the debate of definitions of tumor deposit in recent versions of TNM staging systems. All data were independently proofread three times by Dr. Jun Li, Dr. Xiangxing Kong and Dr. Yibo Cai to ensure accuracy.

### Ph-test of the database

The proportional hazards test (Ph-test) was performed on SEER database (training set) to make sure the hazard ratio of T and N stage was consistent with increasing survival months. Generally, nomogram is constructed based on Cox proportional hazards model or Logistic regression analysis [6]. Therefore, If the database could not pass the Ph-test, Logistic regression model will be used instead of Cox proportional hazards model.

### Construction of nomogram and Nomo-staging system

Fifty percent of patients from the SEER database were randomly classified into a training set while the remainders were classified into a testing set. Combining each T stage and N stage, we developed 25 groups of TN combinations. Chi-squared test was performed between the two data sets to verify the balance of the distribution of 25 TN combinations and the distribution of cancer sites. In the three Logistic regression analyses for construction of nomogram and Nomo-staging system, the endpoint events were 1-year, 2-year and 3-year disease specific survival status respectively. The survival status was defined as 0 for alive, 1 for death due to colorectal cancer and blank for other status. Only T and N stage were included as variables. Three nomograms were then formulated based on the result of Logistic regression analysis [6]. For each TN combination, a total nomo-score was calculated in every nomogram. Each nomo-score corresponded to the survival ratio of relative year. A larger nomo-score represented a poorer prognosis. For each nomogram, normalization of the nomo-score was performed using the formula:

$$ \frac{x-\mathit{\max}}{\mathit{\max}-\mathit{\min}} $$

x represented a specific nomo-score, max represented the highest nomo-score in this nomogram, while min represented the lowest. The average standardized nomo-score of each TN combination from the three nomograms was then calculated. After ranking the average standardized nomo-score, we planned to divide the 25 TN combinations into five groups (I, II, IIIa, IIIb and IIIc) to be in consistence with previous studies [4, 5]. As we found 5 stage groups would make the staging model simplest to analyze while still retain the survival paradox. A group of clinical colorectal oncologists discussed each stage’s cut-off and voted for the Nomo-staging system based on clinical experience and average nomo-score.

### Evaluation of the performance of staging systems

The assessment of the performance of the prognostic system was based on a comprehensive estimation, as we previously described [5], that included: 1) homogeneity, the smaller differences in survival among patients in the same stage indicated a better staging system; 2) discriminatory ability, the greater differences in survival among patients in different stages indicated a better staging system; 3) monotonicity of gradients, the phenomenon that prognosis of patients with earlier stages was better than the patients with higher stages indicated a better staging system.

The Logistic regression analysis on 1-year, 2-year, 3-year overall survival and disease specific survival was performed using the testing set. The likelihood ratio (L.R.) χ2 test was used to measure homogeneity. Staging systems with higher chi-square values are better than those with lower chi-square values. The Akaike information criteria (AIC) value and the concordance index (c-index) were calculated to measure discriminatory ability [12]. A smaller AIC value indicated a better staging system. A higher c-index value indicated a better staging system. Survival curves were drawn using the Kaplan-Meier method. In drafting the Kaplan-Meier curves, the survival status was defined 1 for death caused by colorectal cancer and 0 for other status for disease specific survival, the survival status was defined 1 for death caused by any reasons and 0 for censored data for overall survival. The log-rank test and trend χ2 test were used to determine significance and to compare the discriminatory and gradient monotonicity. A higher χ2 score indicated a better staging system.

### Statistic software

All data was stored in Microsoft EXCEL. R 3.2.2 (GUI 1.66) was used to perform the Ph-test, construct nomograms and perform evaluations. Graphpad Prism 6.0c (GraphPad Software Inc., San Diego, CA, USA) was used to perform Chi-squared tests and draw histograms. The Kaplan-Meier survival curves were drawn using Stata 12.0 software (StataCorp LP, College Station, TX, USA). The distributions of the measurement data were tested by skewness and kurtosis normality tests. Data that was not normally distributed were described by the median and inter-quartile range (M, IQR). A two-sided *P*-value of 0.05 or less was considered to indicate statistical significance.

## Results

### Basic information of the patients from SEER database and SAHZU

A total of 115,377 patients were extracted from the SEER database. The baseline characters were listed in Additional file 1: Table S1. The median follow-up was 39 months (IQR = 51 months). These patients were randomly grouped as training and testing set by ratio 1:1. The distribution of 25 TN combination between the training set and testing set had no significant difference (χ2 = 20.28, *P* = 0.6806, Additional file 2: Figure S1). Additionally, there was no significant difference in the distribution of cancer site between two data sets ($\mathcal{X}$^2^ = 0.0467, *P* = 0.8289, Additional file 2: Figure S1).

A total of 1194 patients were enrolled from the SAHZU database. The baseline characters were listed in Additional file 1: Table S2. The median follow-up was 56 months (IQR = 25 months).

### The construct of the three nomograms

**Fig. 1:**
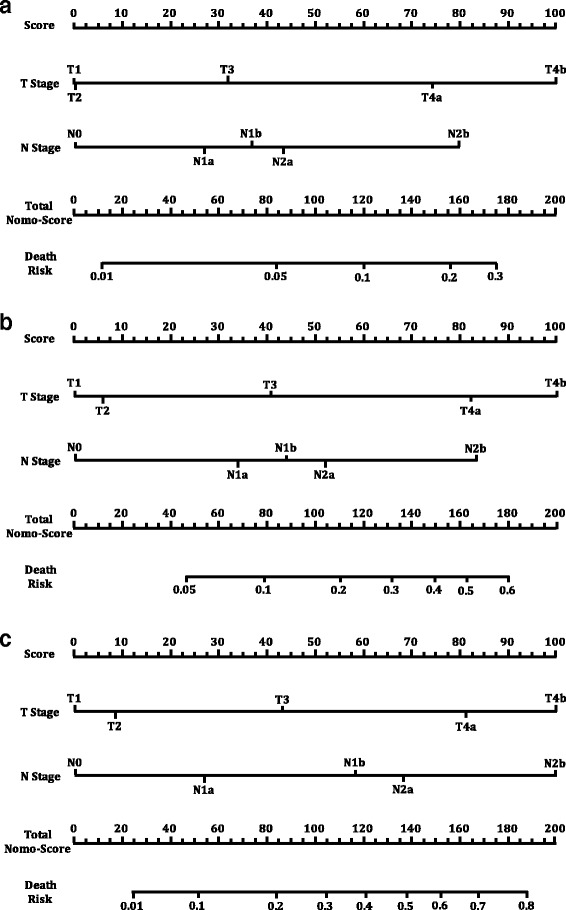
The nomograms of disease specific survival for training set. **a**, 1-year disease specific survival; **b**, 2-year disease specific survival; **c**, 3-year disease specific survival

Three nomograms describing 1 year, 2-year and 3-year disease specific survival were established by R (Fig. 1a-c). Each TN combination had a nomo-score, which indicated the risk of death. The lowest nomo-score was 0 (T1 N0) in 1-year, 2-year and 3-year. This combination indicated the best prognosis, which meant having lowest risk of death in the 1st year, 2nd year and 3rd year post potentially curative surgery. The highest nomo-score was 179 (T4bN2b) in 1-year, 183 (T4bN2b) in 2-year and 198 (T4bN2b) in 3-year. As the median follow-up was only 39 months, the Logistic regression analysis for more than 3-year disease specific survival was impossible.

### Establishment of the Nomo-staging system

The result of ranking average nomo-score was listed in Additional file 1: Table S3. The Nomo-staging system was established according to the expert consensus (Table 1). The distribution of patients in the 7th AJCC TNM staging system and nomo-staging system was listed in Additional file 1: Table S1.

**Table 1:** TN categories of two staging systems for colorectal cancer

Stages	7th TNM staging	Nomo-staging
I	T1-2 N0	T1-2 N0
II	T3-4bN0	T3 N0
		T1-2 N1
		T1N2a
IIIa	T1-2 N1	T4aN0
	T1N2a	T3 N1
		T2N2a
		T1-2N2b
IIIb	T3-4aN1	T4bN0
	T2-3N2a	T4aN1
	T1-2N2b	T3 N2
IIIc	T4bN1	T4bN1
	T4 N2a	T4 N2
	T3-4N2b	

### Evaluation of the Nomo-staging system

**Fig. 2:**
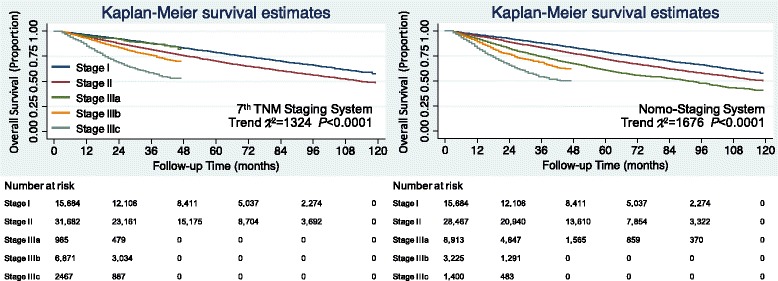
The Kaplan-Meier overall survival curves of testing set colorectal cancer patients according to two staging systems

**Fig. 3:**
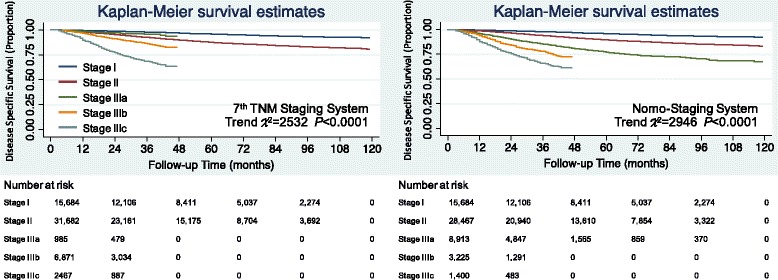
The Kaplan-Meier disease specific survival curves of testing set colorectal cancer patients according to two staging systems

The overall survival and disease specific survival Kaplan-Meier curves in the testing set were constructed. For the Nomo-staging system, stage II and stage IIIa were clearly differentiated for both overall survival (Fig. 2) and disease specific survival (Fig. 3), and the patients with higher stages showed poorer prognoses. For the Nomo-staging system, none of the survival curves for any of the stages crossed. However, for the 7th edition TNM staging system, the overall survival and disease specific survival curves of stage IIIa crossed with stage I which is the reason for the survival paradox. The trend χ2 of the 7th edition TNM staging system was lower than Nomo-staging system, which indicated the improvement of discriminatory ability and monotonicity of gradients.

**Fig. 4:**
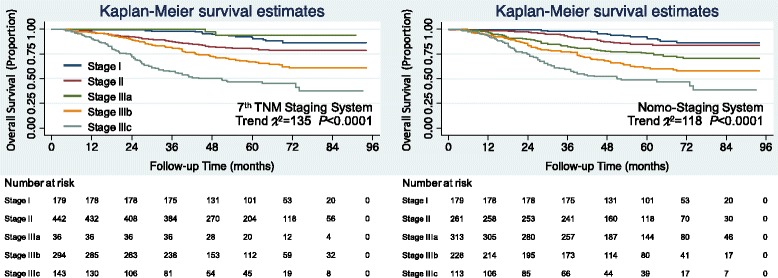
The Kaplan-Meier overall survival curves of SAHZU database colorectal cancer patients according to two staging systems

The external validation of SAHZU database by Kaplan-Meier curves clearly showed the survival paradox between stage II and stage IIIa in the 7th edition TNM staging system (Fig. 4), while the nomo-staging system revised the monotonicity of gradients.

The Logistic regression analysis of the testing set on overall survival (Table 2) and disease specific survival (Table 3) showed the Nomo-staging systems had better homogeneity and discriminatory ability. For 1-year, 2-year and 3-year overall survival, the Nomo-staging system showed higher value in both L.R. (5.9033 e^2^, 1.4835 e^3^, 2.2706 e^3^). and c-index (6.0841 e^−1^, 6.2550 e^−1^, 6.3344 e^−1^). It also showed the lower value in AIC (23,249.95, 34,904.74, 39,082.20). It is also analogous for 1-year, 2-year and 3-year disease specific survival in L.R., c-index and AIC. However, these three indexes did not perform well in the SAHZU database (Additional file 1: Table S4).

**Table 2:** Comparison of the predictive performance of 2 staging systems for overall survival of testing set

Overall survival	Characteristics	7th TNM Staging	Nomo-staging
1-year	c-index	5.8751 e^−1^	6.0841 e^−1^
	AIC	23,403.22	23,249.95
	L.R.	4.4306 e^2^	5.9033 e^2^
2-year	c-index	6.0633 e^−1^	6.2550 e^−1^
	AIC	35,187.99	34,904.74
	L.R.	1.2062 e^3^	1.4835 e^3^
3-year	c-index	6.1699 e^−1^	6.3344 e^−1^
	AIC	39,280.45	39,082.20
	L.R.	2.0783 e^3^	2.2706 e^3^

Abbreviation: *AIC* Akaike information criteria; *L.R* Likelihood ratio

**Table 3:** Comparison of the predictive performance of 2 staging systems for disease specific survival of testing set

Disease specific survival	Characteristics	7th TNM staging	Nomo-staging
1-year	c-index	6.6244 e^−1^	7.0510 e^−1^
	AIC	10,819.46	10,621.42
	L.R.	5.4271 e^2^	7.4074 e^2^
2-year	c-index	6.6396 e^−1^	7.0079 e^−1^
	AIC	18,959.71	18,699.34
	L.R.	1.0801 e^3^	1.3344 e^3^
3-year	c-index	6.4770 e^−1^	6.8123 e^−1^
	AIC	24,244.46	24,015.26
	L.R.	1.2132 e^3^	1.4364 e^3^

Abbreviation: *AIC* Akaike information criteria; *L.R* Likelihood ratio

## Discussion

The existing 7th edition TNM staging system is used to guide to predict the prognosis of patients with colorectal cancer and to guide to discussion of adjuvant treatment [13]. The 8th edition would be officially published in 2018. The preview showed there was no change in the classification of non-metastatic patients. The 8th edition gave a more explicit definition of tumor deposit and defined peritoneal metastasis as M1c. In addition, several biomarkers were unobtrusively recommended to assess patients’ prognosis, but the lack of quantitative detection approach limited its combination with TNM staging system.

The main function of the present staging system was treatment guidance. For example, all stage I patients and some stage II patients with low recurrence risk don’t require adjuvant chemotherapy, while stage III patients are strongly advised to receive postoperative adjuvant chemotherapy [14]. The survival paradox might lead oncologists to overestimate the prognosis risk of stage IIIa, while underestimating the risk of stage II. The cause of this paradox was unclear. Our previous research showed the possible reason for survival paradox in colorectal cancer was the over-weighting of N stage. Therefore, we proposed the T-plus system which enhanced the weighting of T stage, revised the correspondence of stages with TN combinations and eliminated the survival paradox [4]. The T-plus staging system reflects the significance of the T stage in colorectal cancer and abandons the rigid classification according to lymph node status. Additionally, it performed well in a Chinese colorectal cancer retrospective cohort [5].

Here we used nomograms to verify the core concept of T-plus staging system that put T stage weighting higher. Nomograms are regarded as a type of machine learning which gives the computer the ability to learn without being explicitly programmed. It acts more like an auto-grouping machine rather than a simple linear regression analysis. Nomograms use Cox proportional hazards model or Logistic regression model to make cancer survival analysis [15]. In the present study, the training set failed to pass the Ph-test. Therefore, Logistic regression model rather than Cox proportional hazards model was used [6].

Similar to T-plus staging system, not all patients with positive lymph nodes were classified as stage III in the Nomo-staging system. Nomo-staging systems had better performance in prognosis prediction than the 7th edition TNM staging system. Although the core concept of T-plus staging system and Nomo-staging system was consistent, there were still some differences. T-plus staging system strengthened more weighting of T stage than that in Nomo-staging system. For example, in T-plus staging system T1N1a was classified into stage I. In the Nomo-staging system, T1-2 N0 was kept into stage I while T1 N1-2a, T2 N1 were grouped into stage II. Hence, the Nomo-staging system was a more moderate change to the 7th edition TNM staging system comparing to the drastic changes made by T-plus staging system.

The present study did not perform stratified analysis according to the adjuvant treatment because of the limitation of SEER database. Therefore, the obvious debate is that these changes that shift the correspondence of stages and TN combinations should be attributed to the contribution of adjuvant chemotherapy. Over the past twenty years, the progress of adjuvant chemotherapy and radiotherapy had significantly improved the survival of patients with stage III colorectal cancer [16, 17]. The benefits due to adjuvant therapy might narrow the survival gap between stage II and stage III. However, it seemed impossible to thoroughly neutralize the survival difference between stage II and stage III, let alone improve the survival of stage III equal to stage I by adjuvant therapy. For colon cancer, it has been reported the survival paradox was not caused by adjuvant chemotherapy through analyzing U.S. National Cancer Data Base [18, 19]. Considering stages II and III rectal cancer were classified as locally advanced and usually received the same adjuvant chemo-radiotherapy regimen, stage IIIa patients still showed better a prognosis than stage II patients [1, 4]. This indicated that the paradox was derived from the 7th edition TNM staging system instead of adjuvant therapy. Additionally, the SUNRISE study reported stage II/III patients with low 12-gene recurrence score could safely avoid chemotherapy [20]. This study found the heterogeneity of recurrence risks in stage III as well as in stage II colon cancer. As reported, patients with stage II disease in the Recurrence Score high-risk group had a 5-year risk of recurrence similar to patients with stage IIIa/b disease in the low-risk group. This result testified that some stage III patients had a good prognosis and did not require adjuvant chemotherapy. We believe more evidence are necessary to support the argument that these stage III patients do not require adjuvant chemotherapy and should be considered to be reclassified into earlier stages.

There were several limitations in our research. Patients with N1c were combined with N1b in this study. N1c represented tumor deposits found in the pathological specimen. However, the diagnosis criteria of tumor deposits was not consistent considering the criteria kept changing in the recent versions of TNM staging systems [21, 22]. Mayo E. at el showed that tumor deposits were associated with worse 3-year overall survival in patients of any known and unknown N categories [23]. The value of tumor deposits should be assessed in future staging system. The second limitation of this study was that the longest follow-up in the SEER database was only 119 months, and the median follow-up was less than 4 years. Therefore, 5-year overall survival and disease specific survival were not possible to calculated by Logistic regression. Three-year disease specific survival and overall survival were not strong enough endpoints for such an oncological study. Additionally, the lack of treatment information, limited by the current database, might also reduce the reliability of Nomo-staging system. Therefore, we performed external validation on SAHZU database. Although the L.R., c-index and AIC of nomo-staging system did not perform steadily better than the 7th AJCC staging system, the Kaplan-Meier curves showed the survival paradox was revised by nomo-staging system. However, the results should still be verified by other databases with long-term follow-up data and intact treatment information. The third shortcoming was that the Nomo-staging system was only separated to five groups because of the convenience when comparing the performance of the two staging systems by the same number of subgroups. To predict colorectal cancer prognosis more precisely and individually, more subgroups were needed. Moreover, studies have shown that molecular types could predict the prognosis of colorectal cancer independent of the TNM staging system [24–26]. Integrating the prognostic molecular markers, such as microsatellite instability, Ras and Braf gene mutational status and immune-score etc., into the prognosis prediction system may predict the prognosis of colorectal cancer more precisely. Additionally, advanced technology related computer science and big data science should be applied to mine such complex data and to produce a new generation colorectal cancer prognosis prediction system. In addition, the fundamental goal of construction nomo-staging system was to verify the concept of reconsidering the weight of lymph nodes status in prognosis prediction, instead of directly applying nomo-staging system into clinical practice.

## Conclusion

The present study established a modified TNM staging system via nomogram analysis which performed better than the 7th edition TNM staging system in predicting survival of non-metastasis colorectal cancer, which was both validated in SEER testing set and SAHZU database. The Nomo-staging system indicated the weight of lymph nodes status in prognosis prediction should be cautiously reconsidered. However, the robust and rationality of increasing T-stage weight in staging system should be validated in more database considering the limitation of short follow-up time.

## Additional files

Additional file 1: **Table S1.** The Demographic Information of the Patients from SEER Database Enrolled in This Study [online only]. **Table S2.** The Demographic Information of the Patients from SAHZU Database Enrolled in This Study [online only]. **Table S3.** The Ranking of Average Nomo-score [online only]. **Table S4.** Comparison of the Predictive Performance of 2 Staging Systems for Overall Survival of SAHZU dataset [online only]. (DOCX 78 kb)

Additional file 2: Figure S1. The distribution of 25 TN combinations (left) and the distribution of colon cancer and rectum cancer (right) between the training set and testing set [online only]. (TIFF 2963 kb)

## Abbreviations

AIC: Akaike information criteria
AJCC: American Joint Committee on Cancer
c-index: Concordance index
CRC: Colorectal cancer
DSS: Disease specific survival
IQR: Inter-quartile range
L.R.: Likelihood ratio
OS: Overall survival
Ph-test: Proportional hazards test
SAHZU: Second Affiliated Hospital of Zhejiang University
SEER: Surveillance, Epidemiology, and End Results
TNM: Tumor-node-metastasis
