# Supplementary material for: A modified TNM staging system for non-metastatic colorectal cancer based on nomogram analysis of SEER database
Source: BMC Cancer. 2018 Jan 8;18:50. doi: 10.1186/s12885-017-3796-1 (PMC5759792; doi:10.1186/s12885-017-3796-1)
Supplement: Supplementary file 1 — Table S1. The Demographic Information of the Patients from SEER Database Enrolled in This Study [online only]. Table S2. The Demographic Information of the Patients from SAHZU Database Enrolled in This Study [online only]. Table S3. The Ranking of Average Nomo-score [online only]. Table S4. Comparison of the Predictive Performance of 2 Staging Systems for Overall Survival of SAHZU dataset [online only]. (DOCX 78 kb) [file 12885_2017_3796_MOESM1_ESM.docx]

**Supplement Tables**

**Table S1. The Demographic Information of the Patients from SEER Database Enrolled in This Study [online only]**

| **Variable** | **Number** | | **Proportion (%)** | |
| --- | --- | --- | --- | --- |
| **Age** | |  | |  |
| 10-34 | | 1122 | | 0.97 |
| 35-59 | | 28771 | | 24.94 |
| 60-79 | | 57792 | | 50.09 |
| 80-84 | | 14722 | | 12.76 |
| 85+ | | 12970 | | 11.24 |
| **Sex** | |  | |  |
| Female | | 57570 | | 49.90 |
| Male | | 57807 | | 50.10 |
| **Race** | |  | |  |
| White | | 93720 | | 81.23 |
| Black | | 11864 | | 10.28 |
| Other (AI/AN, API) | | 9389 | | 8.14 |
| Unknown | | 404 | | 0.35 |
| **Year of Diagnosis** | |  | |  |
| 2004-2008 | | 51448 | | 44.59 |
| 2009-2013 | | 63929 | | 55.41 |
| **Primary Site** | |  | |  |
| Colon | | 103018 | | 89.29 |
| Rectum | | 12359 | | 10.71 |
| **Histotype** | |  | |  |
| Adenocarcinoma | | 101442 | | 87.92 |
| Mucinous adenocarcinoma | | 10573 | | 9.16 |
| Mucin-producing adenocarcinoma | | 2065 | | 1.79 |
| Signet ring cell carcinoma | | 892 | | 0.77 |
| Undifferentiated carcinoma | | 315 | | 0.27 |
| Mucinous cyst-adenocarcinoma | | 90 | | 0.08 |
| **7^th^ edition TNM Staging System** | |  | |  |
| I | | 31186 | | 27.03 |
| II | | 63452 | | 55.00 |
| IIIa | | 1965 | | 1.70 |
| IIIb | | 13857 | | 12.01 |
| IIIc | | 4917 | | 4.26 |
| **Nomo-Staging System** | |  | |  |
| I | | 31186 | | 27.03 |
| II | | 57021 | | 49.42 |
| IIIa | | 17853 | | 15.47 |
| IIIb | | 6534 | | 5.66 |
| IIIc | | 2783 | | 2.41 |

Abbreviation: AI/AN, API, American India/AkNative, Asian/Pacific Islander.

**Table S2. The Demographic Information of the Patients from SAHZU Database Enrolled in This Study [online only]**

| **Variable** | **Number** | | **Proportion (%)** | |
| --- | --- | --- | --- | --- |
| **Age** | |  | |  |
| 10-34 | | 24 | | 2.19 |
| 35-59 | | 453 | | 41.41 |
| 60-79 | | 553 | | 50.55 |
| 80-84 | | 52 | | 4.75 |
| 85+ | | 11 | | 1.01 |
| Unknown | | 1 | | 0.09 |
| **Sex** | |  | |  |
| Female | | 432 | | 39.49 |
| Male | | 662 | | 60.51 |
| **Year of Diagnosis** | |  | |  |
| 2005-2008 | | 426 | | 38.94 |
| 2009-2011 | | 668 | | 61.06 |
| **Primary Site** | |  | |  |
| Colon | | 597 | | 54.57 |
| Rectum | | 497 | | 45.43 |
| **7^th^ edition TNM Staging System** | |  | |  |
| I | | 179 | | 16.36 |
| II | | 442 | | 40.40 |
| IIIa | | 36 | | 3.29 |
| IIIb | | 294 | | 26.87 |
| IIIc | | 143 | | 13.07 |
| **T-plus Staging System** | |  | |  |
| I | | 52 | | 4.75 |
| II | | 388 | | 35.47 |
| IIIa | | 416 | | 38.03 |
| IIIb | | 229 | | 20.93 |
| IIIc | | 9 | | 0.82 |
| **Nomo-Staging System** | |  | |  |
| I | | 179 | | 16.36 |
| II | | 261 | | 23.86 |
| IIIa | | 313 | | 28.61 |
| IIIb | | 228 | | 20.84 |
| IIIc | | 113 | | 10.33 |

**Table S3. The Ranking of Average Nomo-score [online only]**

| **TN Combinations** | **Average Nomo-score** | **Nomo-staging** |
| --- | --- | --- |
| T1N0 | 0.00 | Stage I |
| T2N0 | 2.61 |  |
| T1N1a | 18.80 | Stage II |
| T3N0 | 20.48 |  |
| T2N1a | 21.40 |  |
| T1N1b | 24.48 |  |
| T2N1b | 27.09 |  |
| T1N2a | 29.10 |  |
| T2N2a | 31.70 | Stage IIIa |
| T3N1a | 39.28 |  |
| T4aN0 | 42.44 |  |
| T3N1b | 44.96 |  |
| T1N2b | 46.66 |  |
| T2N2b | 49.27 |  |
| T3N2a | 49.58 | Stage IIIb |
| T4bN0 | 53.34 |  |
| T4aN1a | 61.24 |  |
| T4aN1b | 66.93 |  |
| T3N2b | 67.14 |  |
| T4aN2a | 71.54 | Stage IIIc |
| T4bN1a | 72.13 |  |
| T4bN1b | 77.82 |  |
| T4bN2a | 82.43 |  |
| T4aN2b | 89.11 |  |
| T4bN2b | 100.00 |  |

**Table S4. Comparison of the Predictive Performance of 2 Staging Systems**

**for Overall Survival of SAHZU dataset[online only]**

| **Overall survival** | **Characteristics** | **7^th^ TNM Staging** | **Nomo-staging** |
| --- | --- | --- | --- |
| 1-year | c-index | 6.7610 e^-1^ | 6.8464 e^-1^ |
|  | AIC | 314.07 | 316.51 |
|  | L.R. | 1.9276 e^1^ | 1.6838 e^1^ |
| 2-year | c-index | 7.2122 e^-1^ | 7.4351 e^-1^ |
|  | AIC | 628.55 | 624.05 |
|  | L.R. | 7.7265 e^1^ | 8.1769 e^1^ |
| 3-year | c-index | 6.1699 e^-1^ | 6.3344 e^-1^ |
|  | AIC | 861.83 | 873.95 |
|  | L.R. | 1.1345 e^2^ | 1.0133 e^2^ |

Abbreviation: AIC, Akaike information criteria; L.R., Likelihood ratio.
