# Supplementary figures and images for: A modified TNM staging system for non-metastatic colorectal cancer based on nomogram analysis of SEER database
Source: BMC Cancer. 2018 Jan 8;18:50. doi: 10.1186/s12885-017-3796-1 (PMC5759792; doi:10.1186/s12885-017-3796-1)

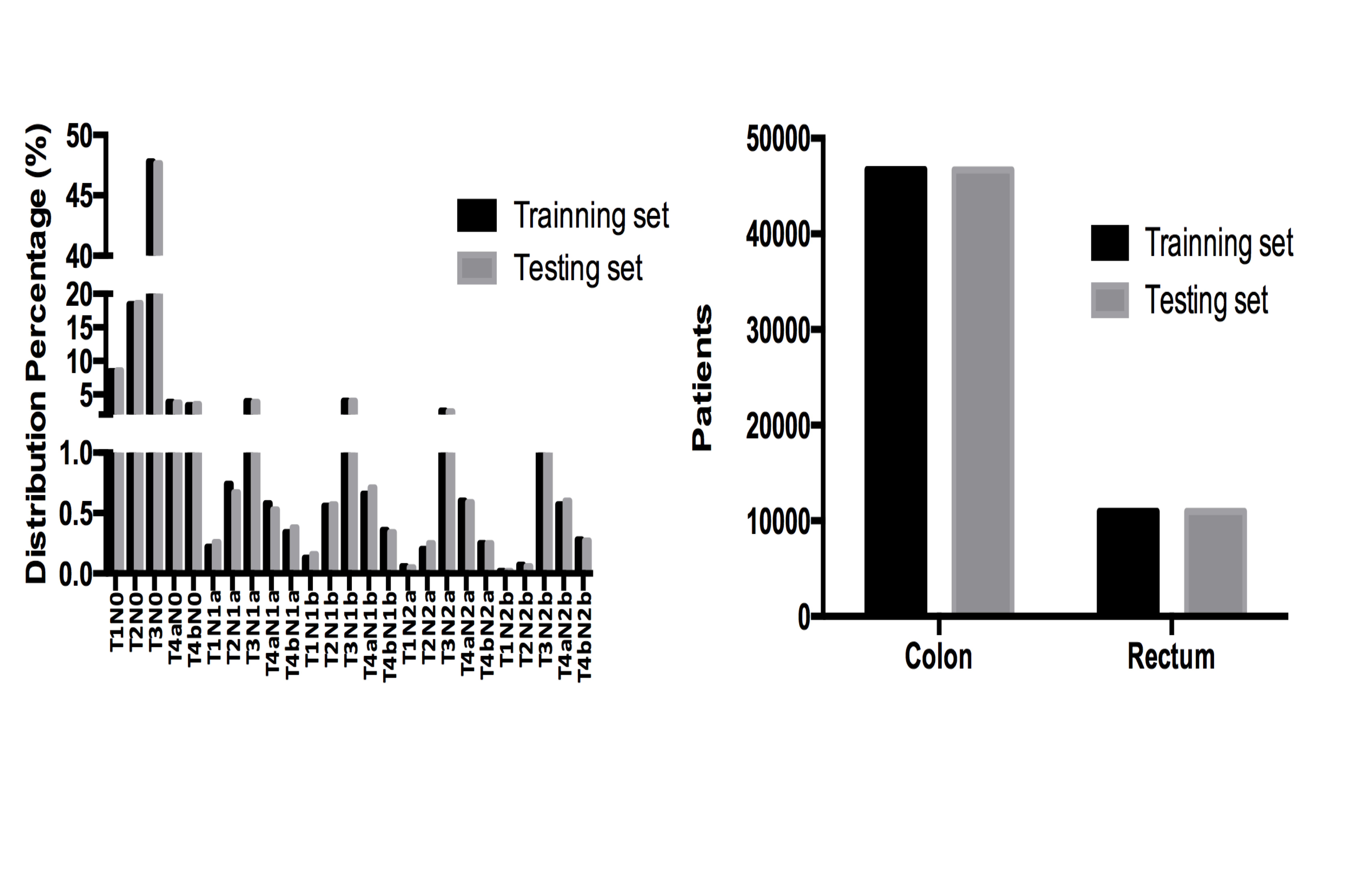

Supplement: Supplementary file 2 — The distribution of 25 TN combinations (left) and the distribution of colon cancer and rectum cancer (right) between the training set and testing set [online only]. (TIFF 2963 kb) [file 12885_2017_3796_MOESM2_ESM.tiff]
